# Hungry for Two Years*In the three preceding articles the editor has departed from his custom of admitting into the Journal of Health only such pieces as he has written himself; but the reader will excuse this as it is made the occasion of inculcating useful lessons and impressing them on the mind by historical facts of a striking character.

**Published:** 1861-07

**Authors:** 


### HUNGRY FOR TWO YEARS.*

SOME men are great in one thing only; others in many; of this latter was the author of the *Cause and Cure of Infidelity*, who was originally a physician of great ability; so much so that his practice became in a short time worth some thousands a year in a community where a single thousand dollars was considered a large sum of money. He subsequently became more distinguished as a speaker than he had been as a doctor. There are thousands yet living in and near New-York city who vividly remember the power of his eloquence, and what crowds flocked to hear him at the old Broadway Tabernacle, the building being filled to its utmost capacity night after night for weeks in succession. He had a herculean frame, and gave many a proof before his embrasure of Christianity, of personal courage and fear-

* In the three preceding articles the editor has departed from his custom of admitting into the Journal of Health only such pieces as he has written himself; but the reader will excuse this as it is made the occasion of inculcating useful lessons and impressing them on the mind by historical facts of a striking character.

lessness, but he was an epilectic; and he knew that the tendencies of his system were in that direction, and that the only method of preventing the attacks was the most rigid control of the appetite. His medical knowledge placed this truth so distinctly before his mind, and his self-control was so heroic, that in speaking of it on one occasion to those very dear to him, he said: "I have been hungry for two years." This was exhibiting a rational self-denial worthy of all admiration, and is a lesson to that vast multitude which no man can number who have not enough force of character, of moral courage, to prevent their yielding to their appetites three times a day, not merely to the extent of satiety, but actual repletion, really eating, and that habitually more than nature needs. Man is like a pig; when he is hungry he gets quarrelsome, ill-natured, snappish; but in one respect he is unlike a hog; that animal with all its love of filth, ceases to eat when it is no longer hungry. Reader, did you never eat to make it even? take some more bread because you had a little butter on your plate, or take a little more sauce, or gravy, (called "essence" in the higher spheres) because a bit of bread was left, and you did not want to leave it. That was waste; it was worse than waste, because it not only did you no good, but a positive injury. The great name mentioned in this article under the influence of the gnawings of hunger, voluntarily endured, was one of the loveliest men in his disposition, we ever encountered; for we once lived under the same roof with him, and personally know of what we speak. The narration has been given in order to enforce a lesson on the subject of EPILEPSY, which is so often engendered by injudicious eating; an attack often arising from a single injudicious meal, in very young children, and being repeated several times in succession, becomes a habit to afflict through life, wearing out the intellect by slow degrees, ending in hopeless imbecility, if not more speedily by a "fit," falling from a horse, or vehicle, or into a river, or into the fire. But from whatever cause, Epilepsy is more certainly kept under control indefinitely by a rigid system of dieting than in any other way. Dr. Nelson proved this in his own case, and died in the full possession of all his giant faculties.

